# Climate Change and Environmental Influence on Prevalence of Visceral Leishmaniasis in West Pokot County, Kenya

**DOI:** 10.1155/2022/1441576

**Published:** 2022-09-20

**Authors:** Bulle Abdullahi, Joshua Mutiso, Fredrick Maloba, John Macharia, Mark Riongoita, Michael Gicheru

**Affiliations:** ^1^Department of Community Health and Epidemiology, School of Public Health, Kenyatta University, Nairobi, Kenya; ^2^Department of Zoological Sciences, School of Pure and Applied Sciences, Kenyatta University, Nairobi, Kenya; ^3^Ministry of Health, County Government of West Pokot, Kapenguria, Kenya; ^4^Department of Tropical and Infectious Diseases, Institute of Primate Research, Nairobi, Kenya

## Abstract

Kala-azar is a parasitic disease caused by *Leishmania species* transmitted by sand fly. In Kenya, kala-azar is endemic in thirty subcounties spread over in eleven counties in the arid zones. Climate change-influenced seasonal weather variability and environmental alterations remain important determinants of many vector-borne diseases. The present study focused on climate change and environmental influence on kala-azar in West Pokot. A descriptive cross-sectional and retrospective research design was adapted. Study area was purposively selected. Locations were randomly selected, and households were systematically selected. Three hundred sixty-three household questionnaires, eleven key informant interviews, and five focus group discussions were undertaken. Secondary data were obtained from Kacheliba subcounty hospital records. Statistical Package for the Social Sciences version 24 was used to analyze quantitative data while qualitative data were analyzed to establish connection for interpretation. Kala-azar cases have been on the rise on aggregate and surge towards the end of dry season and just after the rains. Significant environmental factors included the presence of seasonal rain water pathways and rock piles around houses (AOR = 4.7; 95% CI = (2.3-9.6), *p* < 0.05), presence of acacia trees in and around homesteads (AOR = 8.5; 95% CI = (2.5-28.6), *p* < 0.05), presence of anthills around the homesteads (AOR = 5.2; 95% CI = (1.2-23.4), *p* < 0.05), and presence of animal shed within compound (AOR = 2.8; 95% CI = (0.96-8), *p* < 0.05). Climate change-induced seasonal weather variability, increased temperature and reduced precipitation as well as environmental alterations influence kala-azar occurrence in West Pokot. Community sensitization on disease prevalence, clearing of vector predilection sites, and improving community environmental risk perception are imperative to promote prevention.

## 1. Introduction

Leishmaniasis is a vector-borne protozoan re-emerging disease caused by *Leishmania species* and transmitted by sand fly vectors [1]. Leishmaniasis is a neglected disease receiving little development and research investments, thus, is considered as a disease of poverty as majority of its victims are people with humble economic background who reside at the remote areas with limited basic resources like health care [2, 3]. Leishmaniasis has four major disease forms which range from simple skin lesions to fatal systemic infection and include cutaneous form, visceral form, muco-cutaneous form, and postkala-azar dermal leishmaniasis form [4, 5]. Visceral leishmaniasis is considered most lethal among all the forms with over 95% fatality rate if untreated and second most killer parasitic disease after malaria [3, 6, 7]. Although only 25-45% of the incidents are reported, visceral leishmaniasis is endemic in approximately hundred countries worldwide and more than ninety percent of the cases occur in ten countries mainly in Eastern Africa, South-East Asian region, and Brazil [3]. Globally, the prevalence of leishmaniasis is approximately 12 million and three hundred fifty million people are at risk of infection [8]. Visceral leishmaniasis remains to be the most lethal form with a prevalence of approximately four million, between 50,000 and 90,000 annual new cases, and about 50,000 annual fatalities worldwide [5, 9, 10].

After the Indian subcontinent, Eastern Africa is considered to be contributing second highest number of visceral leishmaniasis cases and they are mainly caused by *Leishmania donovani* [11, 12]. In Kenya, visceral leishmaniasis was first documented in early 19^th^ century when an outbreak occurred around Lake Turkana area [13]. Currently, in Kenya, visceral leishmaniasis is endemic in over eleven counties which are reporting approximately one thousand five hundred cases annually, and more than five million people are at risk of this disease mostly in the arid and semiarid climatic zones [4, 14, 15]. Considering the fast setting-in of climate change effect which triggers frequent alterations on seasonal weather pattern, both the global prevalence and emergence of vector-borne diseases in uncommon areas are expected to rise [16]. Regardless of phlebotomine species involved and reservoir behaviors, weather fluctuations and environmental modifications are considered strong factors that may influence the occurrence of leishmaniasis [17, 18]. Similarly, global warming and climate change-induced alteration in temperature and precipitations variability remain to be significant factors affecting vector epidemiology in terms of abundances and activeness [19]. In African where more than seventy percent of the population lives in rural areas, it is expected that the continent will experience more of the effect of climate change and global warming as a result of the emerging new settlements and widespread alteration of environment through deforestation which may promote creation of more vector breeding sites [20]. Increase in temperature is believed to favor sand fly multiplication while also increasing its period of activity [21]. Environmental factors surrounding homesteads including presences of certain plant species, seasonal rain water pathways-created land fissure and crevices, presence of animal sheds, and chicken houses are thought to influence the presence as well as activity of the vector [18, 22, 23].

With the devastating effect of global warming and climate change coupled with prevalent low individual immune system and social weakness in the visceral leishmaniasis endemic zones, cases of leishmaniasis are expected to get aggravated, thus, the need to undertake epidemiological studies particularly in endemic areas [24–26]. The current study aimed to contribute to the body of knowledge on visceral leishmaniasis in achieving the World Health Organization's neglected tropical diseases road map target 2021 to 2030 and sustainable development goal number 3.3 which target to end neglected topical disease epidemics by 2030, as well as Kenya's ministry of health 2021-2025 strategic plan for control of visceral leishmaniasis and vision 2030. The present study endeavors to ascertain climate change and environmental influence on visceral leishmaniasis in West Pokot County in order to provide essential information to help in upscaling strategies to reduce visceral leishmaniasis burden.

## 2. Materials and Methods

### 2.1. Study Area

The current study was carried out from September 2020 to February 2021 in West Pokot County and particularly in Kacheliba subcounty. The subcounty has 3,910 km^2^ land mass and is home to 134,485 persons [27]. The area experiences temperature of 20°C to 39°C and mostly seasonal rainfall of approximately 500 mm. Kacheliba borders with Turkana County to the north and east and international border with Uganda to the west.

### 2.2. Study Design

The study combined both descriptive cross-sectional and retrospective study design aimed at establishing climate change and environmental alterations-induced seasonal weather variability influence on visceral leishmaniasis burden from 2010 to 2020 in West Pokot County.

### 2.3. Sampling

The study area was selected purposively considering the high number of kala-azar cases reported and presence of visceral leishmaniasis treatment and diagnostic centre. Random sampling of locations and systematic selection of households that lived in the study area for more than six months prior to study commencement was undertaken. Three hundred sixty-three household questionnaires and observation checklist, eleven key informant interviews, and five focus group discussions were carried out and secondary data were obtained from Kacheliba subcounty hospital records. Historical Kacheliba temperature and rainfall amount obtained from world weather online was used as the weather parameter. Sample size was determined by using Cochran's *n* = *Z*^2^*Pq*/*e*^2^ formula.

### 2.4. Data Analysis

Statistical Package for the Social Sciences (SPSS) version 24 and Microsoft Excel software were used to analyze quantitative data, while chi-square and binary logistic regression were performed as bivariate and multivariate analyses. NVivo software was used in performing qualitative data analyzed to establish connection and interpretation in identification of patterns. Correlation analysis between the disease burden and temperature or rainfall amount was performed using Pearson correlation test. Only variables with *p* value of less than 0.05 were considered significant.

## 3. Results

### 3.1. Household Environmental Characteristics

Distribution of environmental characteristics that were considered significant in influencing the risk of visceral leishmaniasis in the study area was cross-tabulated (Table 1). Fifty-three point one percent of the respondents had seasonal rain water pathways or rock piles existing around their homesteads while 46.9% had none of these in close proximity. Almost three quarters of the participants (74.7%) had acacia trees present in and around their compounds while a quarter (25.7%) had either uprooted or lived in areas where this tree species were not commonly grown. A considerable percentage (37.5%) of respondents had garbage dumping sites present within fifty meters proximity to their houses, while the majority (62.5%) reported to dispose waste haphazardly in their farm fields and surrounding community rangeland. Majority (81.7%) of the study participants had anthills present in and around their compounds, while few (18.3) had either uprooted or lived in areas where anthills were not common. More than eighty percent of the household respondents had constructed animal sheds and chicken shelters within their compounds. Considering traditional practice of moving houses to temporary new compounds, 29.2% of the household respondents reported practicing nomadic habit of frequently moving houses from time to time and to different locations.

### 3.2. Household Environmental Characteristics and their Association with Visceral Leishmaniasis

Binary logistic regression was carried out in multivariate analysis to obtain the reduced model, and all variables significant at bivariate analysis were consequently adjusted. After subjecting backward conditional technique on significant environmental variables, only five variables remained statistically significant. The result in Table 1 indicates that members of households who had presence of seasonal rain water channels or rock piles around their homesteads were 4.7 more times likely to get infected by visceral leishmaniasis than those without seasonal water channels and rock piles (AOR = 4.7; 95% CI = (2.3-9.6), *p* < 0.05). The participants who had acacia trees present in and around their houses were 8.5 times more likely to be infected by visceral leishmaniasis (95% CI = (2.5-28.6), *p* < 0.05), as well as those with dumping site around their houses (AOR = 1; 95% CI = (0.5-2.0), *p* < 0.05). Further data analysis indicates that occupants of houses with anthills in and around their compound were 5.2 more likely to be infected with visceral leishmaniasis than those without anthills (AOR = 5.2; 95% CI = (1.2-23.4), *p* < 0.05). Occupants of houses where animal sheds were present in their compound were 2.8 times (95% CI = (0.96-8), *p* < 0.05) more likely to get infected by visceral leishmaniasis. Respondents who practice nomadic culture of moving houses to new temporary compounds had 2 times more chances of getting infected with visceral leishmaniasis than those who reside in permanent compounds (AOR = 2; 95% CI = (1-4), *p* < 0.05; Table 1).

### 3.3. Trends of Kala-Azar Cases, Temperature, and Rainfall in Kacheliba Subcounty

**Figure 1:**
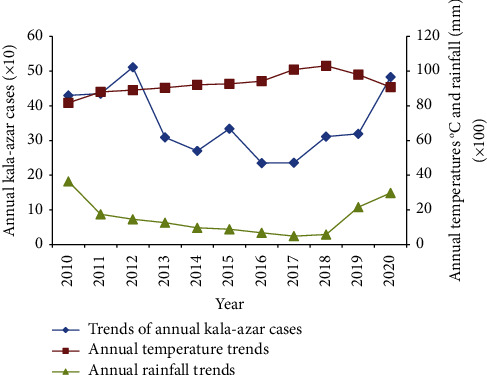
Trends of annual kala-azar cases, temperature, and rainfall from 2010 to 2020 in Kacheliba subcounty.

Figure 1 shows annual visceral leishmaniasis cases as obtained from Kacheliba subcounty hospital records and Kacheliba subcounty annual temperature as well as rainfall trends as obtained from world weather online (https://www.worldweatheronline.com/kacheliba-weather/rift-valley/ke.aspx) for the years 2010 to 2020. The results show overall increase in average daily temperature from 23°C in 2010 to 26°C in 2020 (annually aggregate increase from 8169°C in 2010 to 9100°C in 2020). Similarly, the annual kala-azar cases treated at Kacheliba subcounty hospital tend to have increased on aggregate from approximately four hundred cases in 2010 to almost five hundred in 2020. Regarding the relationship with weather, findings show that annual kala-azar cases and annual temperature trend tend to have weak positive correlation (*r* = 0.609; *P*=0.47). The results further indicate the overall annual rainfall amount in Kacheliba subcounty had reduced from 3643 mm in 2010 to 2976 mm in 2020. Precipitation in Kacheliba subcounty tends to have significant negative correlation with annual kala-azar cases (*r* = −0.7; *P*=0.016).

### 3.4. Monthly Variation of Kala-Azar Cases in Kacheliba Subcounty

**Figure 2:**
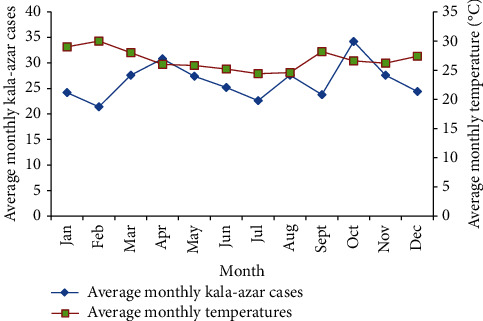
Monthly variations of kala-azar cases as obtained from Kacheliba subcounty hospital records during 2016 to 2020.

Figure 2 shows the number of visceral leishmaniasis cases treated at Kacheliba hospital per month during the last half a decade (2016 to 2020) as obtained from Kacheliba subcounty hospital records. According to the hospital records, cases have been increasing from approximately three hundred in 2016 to slightly under five hundred in 2020. Visceral leishmaniasis cases per month ranged between twenty and approximately thirty-five during this half a decade. Concerning seasonal influence, visceral leishmaniasis cases tend to increase towards the end of first quarter around March and April as well as start of last quarter around October of every year, while remaining relatively low during middle quarter of the year.

## 4. Discussion

Visceral leishmaniasis is endemic in Kacheliba subcounty and remains to be the second most prevalent parasitic disease after malaria, and these findings agree with previous reports that ranked kala-azar as second most lethal parasitic disease [2, 6]. The present findings point out to a multiplex relationship between kala-azar cases and climate change, as an increase in global temperatures and a decrease in rainfall amount tend to significantly influence kala-azar cases and these relationships agree with reports in earlier studies [28–30]. Moreover, the observed surges in kala-azar cases towards the end of dry season as well as just after the rains while remaining relatively low during cold weather season depict the level of interaction between climate change-induced seasonal weather variability and their impact on kala-azar incidents among other vector-borne diseases. The seasonal weather variability influence on vector habits is further demonstrated by the increase in kala-azar cases during low precipitation period. This may be attributed to elevated vector population as well as vector expansion to uncommon areas towards the end of dry seasons. This season is usually characterized by high humidity as well as wider range of temperature variations; these observations support previous reports [21, 30, 31] which indicated a positive correlation between vector abundance and increased temperature variability. Overall, climate change-induced prolonged drought results in depletion of available surface water and rangeland pasture coverage, hence, triggers mass movement of humans and animals into deeper virgin high vector density areas and these findings are in conformity with previous reports [32–35].

The significantly associated environmental factors like existence of seasonal rain water channels, presence of acacia tree, and anthills in and around human houses with high kala-azar infection rates may indicate the complexity of interaction between ecological and lifestyle factors predisposing the communities to disease. Long after rains, big woody plants on the lowland areas particularly along seasonal riverbanks and swamps remain green and provide shades from scotching sun to both herders and their livestock during hot weather dry season. Likewise, these plants as well as riverbank fissures and crevices form humid vector preferred hiding and breeding zones, hence providing potentially risky areas of infection [18, 22]. Moreover, during flooding periods, these seasonal rain water pathways tend to move the sand fly larvae from the endemic zones to new different places, hence, increasing risk of transmission as well as vector expansion to new areas [36]. The moderately humid atmosphere and plenty of hiding areas inside the anthill holes are believed to form perfect resting and breeding environment for the sand fly; thus, its presences around the human houses increase the risk of infections [37]. The flowers and secretions of the acacia tree provide both feed and water to the sand fly, while its bark provides perfect resting and breeding place; hence, presence of acacia in and around the compound poses risk of attracting the vector and exposing those living nearby [23, 37].

Presence of animal sheds and chicken shelter as well as movement of houses from time to time to new temporary compounds and locations were associated with risk of visceral leishmaniasis infection, and this poses complication in disease control as these practices are both economical and cultural in nature. Many times, constructed animals and chicken shelters tend to be wet long after rains creating moistening of the droppings and humid closed environment that are believed to attract the vector while the structure provides resting spaces [38, 39]. Traditional nomadic culture of frequently moving houses and people to new compounds which is commonly practiced by the pastoral community living in the vector endemic arid and semiarid areas is associated with increased risk of infection. The risk is believed to be arising from the high vegetation density in the newly settled temporary compound which may be already infested with the vector or attract it as a result of human activities such as newly cultivated agricultural fields [34, 40, 41]. Our data are inconsistent with earlier report by Macharia et al. 2018, in Baringo, Kenya, in which the community could not associate risk of kala-azar infection and environmental factors [42]. Interestingly, this may possibly exemplify the poor risk perception of the pastoral community on environmental associated risk factors of kala-azar infection. The limitations of present study remain to be those of cross-sectional and retrospective studies as data on household kala-azar cases and environmental risk factors were simultaneously assessed, as well as gaps of facility records data. In conclusion, the impact of environmental factors and climate change-induced seasonal weather pattern variability emerges as important factors that influence vector distribution and habitats as well as number of kala-azar cases in the study area. The study recommends improvement in community risk perception, introduction of culturally acceptable behavior and habitat modification, and reducing vector harboring structures around houses while observing environmental conservation to mitigate the impact of climate change on kala-azar.

## Figures and Tables

**Table 1:** Household environmental characteristics and their association with visceral leishmaniasis in Kacheliba subcounty of West Pokot County, Kenya (*n* = 360).

		Kala-azar present			Adjusted odds ratio (95% CI)		*p*-Values
Variable	Category	Yes	No	Total	AOR	Lower and upper	
Water pathways/rock pile presence							
Yes		63 (17.5%)	128 (35.6%)	191 (53.1%)	4.7	2.3-9.6	<0.05
No		15 (4.2%)	154 (42.8%)	169 (46.9%)			

Acacia tree presence							
Yes		74 (20.6%)	195 (54.2%)	269 (74.7%)	8.5	2.5-28.6	<0.05
No		4 (1.1%)	87 (24.2%)	91 (25.3%)			

Dumping site presence							
Yes		41 (11.4%)	94 (26.1%)	135 (37.5%)	1	0.5-2.0	0.097
No		37 (10.3%)	188 (52.2%)	225 (62.5%)			

Anthill presence							
Yes		76 (21.1%)	218 (60.6%)	294 (81.7%)	5.2	1.2-23.4	<0.05
No		2 (0.6%)	64 (17.8%)	66 (18.3%)			

Animal shed presence							
Yes		70 (19.4%)	221 (61.4%)	291 (80.8%)	2.8	0.9-8.0	0.0599
No		8 (2.2%)	61 (16.9%)	69 (19.2%)			

Chicken shelter presence							
Yes		53 (14.6%)	265 (73.2%)	318 (87.8%)	0.93	0.4-0.2	<0.05
No		25 (6.9%)	19 (5.2%)	40 (12.2%)			

Recently moved house							
Yes		39 (10.8%)	66 (18.3%)	105 (29.2%)	2	1.0-4.0	<0.05
No		39 (10.8%)	216 (60.0%)	255 (70.8%)			

AOR = adjusted odds ratio, binary logistic regression result.

## Data Availability

All data collected and analyzed for this study are included in the article.
